# Newborn Resuscitation Practices and Outcomes in Rural Tanzania—A Real-Time Observational and Video Study

**DOI:** 10.3390/children13050614

**Published:** 2026-04-28

**Authors:** Anita Yeconia Bukhay, Hanne Pike, Joar Eilevstjønn, Raphael Mduma, Ladislaus Blacy, Estomih Mduma, Robert Moshiro, Jackie K. Patterson, Siren Rettedal, Hege Ersdal

**Affiliations:** 1 Haydom Lutheran Hospital, Haydom P.O. Box 9000, Tanzania; raphaelmduma@gmail.com (R.M.); ladisblacy@yahoo.com (L.B.); estomih.mduma@haydom.co.tz (E.M.); 2 Faculty of Health Sciences, Stavanger University, 4036 Stavanger, Norway; hanne.markhus.pike@sus.no (H.P.); siren.irene.rettedal@sus.no (S.R.); 3 Department of Pediatrics, Stavanger University Hospital, 4011 Stavanger, Norway; 4 Laerdal Medical, 4002 Stavanger, Norway; joar.eilevstjonn@laerdal.com; 5 Muhimbili National Hospital, Dar Es Salam 11103, Tanzania; robert.moshiro@mnh.or.tz; 6 Department of Pediatrics, Division of Neonatal-Perinatal Medicine, University of North Carolina, 101 Manning Drive, 4th Floor, CB #7596, Chapel Hill, NC 27599-7596, USA; jackie_patterson@med.unc.edu; 7 Department of Simulation-Based Learning, Stavanger University Hospital, 4011 Stavanger, Norway

**Keywords:** newborn resuscitation, resuscitation guidelines, Helping Babies Breathe, bag-mask ventilation, adherence with guidelines, birth asphyxia

## Abstract

**Highlights:**

**What are the main findings?**

- In this referral low-resource hospital, 10% of newborns received bag-mask ventilation after birth;
- Two-thirds were ventilated after the “golden minute”, often due to excessive use of stimulation and suction;
- Duration of bag-mask ventilation for more than 10 min increased the risk of death significantly.

**What are the implications of the main findings?**

- The complex and urgent situation of newborn resuscitation is difficult to handle according to guidelines;
- Real-time documentation of resuscitations is essential and important to accurately understand real-world practices;
- Prolonged ventilation (>10 min) signals high-risk cases;
- Training and quality-improvement interventions are needed to optimize care.

**Abstract:**

Background: Birth asphyxia is a leading cause of neonatal mortality. More than half of these deaths are due to low-quality care. Objectives: To describe the frequency, sequence, timing, and duration of interventions after birth and newborn outcomes. Methods: This prospective observational study in rural Tanzania included newborns ≥28 weeks gestation. Trained research assistants observed and recorded all deliveries and resuscitations 24 h a day, 7 days a week, logging interventions in real time using the Liveborn Observation app. Results: Of 2564 newborns born, 2431 (94.9%) were enrolled in the study. Macerated stillbirth (*n* = 52), newborns with no parental consent (*n* = 67) or incomplete Liveborn data (*n* = 14) were excluded. Additionally, 2193/2431 (90.2%) newborns did not receive bag-mask ventilation (BMV), and 1755/2431 (72.2%) started breathing before 30 s from birth at median (quartiles) 6 (3, 13) s, 438/2431 (18.0%) started breathing beyond 30 s at 49 (38, 67) s. Moreover, 238/2431 (9.8%) received BMV at 82 (54, 120) s after birth, 1/3 within the first min. Finally, 159/238 (66.8%) were suctioned for 26 (17, 40) s. The first suction sequence was initiated at 44 (24, 78) s after birth. In 24/238 (10.1%) newborns, BMV continued for more than 10 min, with an increased risk of dying within 24 h (RR = 4.26, 95% CI; 1.3–10.0, *p* = 0.016) and seven days (RR = 8.14, 95% CI; 3.5–17.6, *p* < 0.001) compared to those ventilated for less than 10 min. Conclusions: Almost 10% of newborns received BMV at birth, but only one-third were ventilated within the first recommended minute. Excessive use of suctioning likely delayed the start of BMV, and prolonged ventilation beyond 10 min was associated with higher mortality.

## 1. Introduction

Neonatal mortality remains a global challenge, and every year 1 million newborns die within the first 24 h of life, mostly due to complications from birth asphyxia [1,2]. “Birth asphyxia” refers to the inability to maintain an adequate oxygen supply to tissues, resulting from compromised blood flow occurring before, during, or immediately after birth. The primary cause of birth asphyxia is inadequate monitoring and treatment during labor and in the immediate postnatal period [3,4]. Although most women in low- and middle-income countries have physical access to delivery facilities, many of these facilities do not meet adequate quality standards [5].

About 43% of neonatal deaths in 2022 occurred in Sub-Saharan Africa, with Tanzania being among the countries with a high mortality rate at 24 per 1000 live births [1]. More than half of neonatal deaths are preventable with good-quality care [1,2].

In low-resource settings, neonatal resuscitations should follow the Helping Babies Breathe (HBB) guidelines [6] endorsed by the World Health Organization (WHO). HBB is now part of the broader joint program by WHO and the American Academy of Pediatrics, known as Essential Newborn Care (ENC) [3]. Initial implementation of HBB in eight hospitals in Tanzania resulted in a reduction in assumed fresh stillbirths by 24% and 24 h newborn deaths by 47% [7]. Based on this success, HBB training was provided in 15 regions of mainland Tanzania. When tested immediately after the training, 87.1% of healthcare providers passed the objective structured clinical examination; however, only 55.8% passed the same examination when tested after four to six months [8]. HBB guidelines recommend that within the first minute after birth, the newborn should be dried thoroughly, kept warm, and, if not breathing well, stimulated by rubbing the back two or three times, have the airway cleared if needed, and receive bag-mask ventilation (BMV) if spontaneous breathing still has not commenced. Rapid evaluation of chest movements and corrective steps after initiation of BMV are emphasized. HBB recommends uninterrupted ventilation until the newborn is breathing spontaneously, or if the newborn does not start breathing, seek advanced care. The WHO recommends that resuscitation should be discontinued after 10 min if there is no heart rate or after 20 min if there is no spontaneous respiration following effective ventilation [9]. Adherence to newborn resuscitation guidelines remains challenging in both high [10,11,12,13] and low-resource settings [14,15,16,17,18], although timely and good-quality BMV is crucial to save newborn lives [17,19]. Correct and detailed documentation of newborn resuscitations—a very complex and stressed clinical situation—is a common challenge, leading to a persistent lack of high-quality studies to inform best practices after birth [20,21].

In this study we describe the frequency, sequence, timing, and duration of interventions immediately after birth and the relationship between duration of ventilation and newborn outcomes at 24 h and 7 days after birth.

## 2. Materials and Methods

### 2.1. Study Design and Setting

This prospective descriptive observational study, conducted at Haydom Lutheran Hospital, is part of the Safer Births innovation and research project to improve perinatal lives. Haydom is a referral hospital located in the Manyara region in rural northern-central Tanzania. The hospital serves a population of two million people and has a 420-bed capacity, with approximately 3400 births annually. Midwives conduct almost all newborn resuscitations with doctors on call 24 h a day, 7 days of the week. Each delivery room has a newborn resuscitation station equipped with an overhead warmer, the Upright Resuscitator (Laerdal Global Health, Stavanger, Norway), the dry-electrode heart rate monitor NeoBeat (Laerdal Global Health, Stavanger, Norway), the Penguin Newborn Suction (Laerdal Global Health, Stavanger, Norway), and a tablet with a video camera and display (Figures S1 and S2).

At Haydom Lutheran Hospital, it is standard practice to provide annual training on newborn resuscitation skills for all healthcare providers. Newly recruited staff receive this training upon commencement of their duties in the maternity ward, while senior staff participate in refresher training sessions once every year. In addition, healthcare providers engage in regular low-dose, high-frequency simulation training using the NeoNatalie Simulator (Laerdal Global Health, Stavanger, Norway), which is stationed in the labor ward. Each provider is expected to practice at least once per week to maintain and enhance their skills. Some of them have received certificates in Newborn Life Support.

Data collection for this study was from 14 April through 31 December 2023.

### 2.2. Study Population

All newborns born in the hospital at ≥28 weeks gestational age were eligible for enrollment. Macerated stillbirths were excluded, but fresh stillbirths were included since there is a known misclassification between fresh stillbirths and alive, compromised newborns [22]. Newborns with parental consent for participation and complete Liveborn Observation registrations were enrolled in the study.

### 2.3. Data Collection

Trained research observers were present in the maternity ward 24 h a day, 7 days a week. There were three shifts with 2–3 observers on each shift. They continuously extracted maternal and newborn characteristics and labor interventions from the partograms and medical records. Time of birth, newborn breathing status, drying, skin-to-skin contact, time of cord clamping, and resuscitative measures such as suctioning, stimulation and BMV were captured in real-time in the Liveborn Observation app (Laerdal Global Health, Stavanger, Norway) on a tablet by the observers (Figure S2). The dry-electrode heart rate monitor was placed on all newborns immediately after birth if resuscitation was required. Heart rate was transferred to and stored in the Liveborn Observation app. If BMV was needed, the newborn was moved after cord clamping to the nearby resuscitation table. Video cameras above the resuscitation tables captured the newborn and the hands of the healthcare providers. Patients were followed up for 7 days or until discharge/death (whichever came first).

Ethical approval, including maternal and culturally appropriate consent procedures for video recording of newborn resuscitation, was obtained before study initiation.

### 2.4. Definitions

The sequential order of resuscitative interventions according to the HBB guidelines is (i) dry and keep warm, (ii) stimulate if not breathing well, (iii) suction if secretion is obstructing the airway, and (iv) start BMV within one minute if not breathing well. In our study, the observers recorded “drying” if amniotic fluid and blood were thoroughly removed from the baby’s skin using a clean, warm towel right after birth. “Keep warm” was recorded if the newborn was dried, placed skin-to-skin on the mother’s chest or abdomen, and wrapped in a clean, dry cloth covering the head. “Stimulation” was defined as repetitive, tactile rubbing of the newborn’s torso. “Suctioning” was defined as the time from the tip of the suction first being passed into the nostril or mouth until its exit. “Total duration of BMV” was defined as the cumulative number of s of ongoing ventilation. “Prolonged BMV” was defined as ongoing ventilation at 10 min after birth, regardless of the total duration of BMV.

Fresh stillbirths were classified by the midwifes. Early newborn death was defined as a liveborn and dying within 24 h after birth.

### 2.5. Annotation of Videos

Newborn resuscitation videos of cases with prolonged BMV were reviewed and annotated by three research investigators (AY, RM, and HP) using the ELAN 5.8 tool (The Language Archive, Nijmegen, The Netherlands). The videos were first annotated by at least two researchers, and if discrepancies in annotations were found, the videos were reviewed and consensus reached. “Keep warm” was defined as the thorough removal of amniotic fluid and blood from the baby’s skin using a clean and dry cloth before wrapping the newborn in another clean, dry cloth and covering the head. Good team coordination was considered present when at least one team member actively provided instructions or guidance to others.

Finally, a pediatrician (HP) evaluated the quality of BMV. Effective ventilation was defined as chest movements synchronized with the ventilation frequency, without frequent interruptions in ventilation. In cases of ineffective ventilation, defined as no chest rise or continued low heart rate, mask positioning, seal, and head position were annotated. Good head extension was considered maintained when the head was slightly tilted.

### 2.6. Statistical Analysis

Liveborn app registrations and NeoBeat signal data were extracted and analyzed using MATLAB R2024b (MathWorks Inc., Natick, MA, USA) or Microsoft Excel 365 (Microsoft, Redmond, WA, USA). Plots illustrating the frequency, sequence, timing and duration of resuscitative interventions, with heart rate responses, were prepared.

Continuous data were summarized and presented as medians (quartiles); categorical data were presented as count (%). The relative risk (RR) of death versus survival at 24 h and 7 days postpartum, if BMV continued for 10 min or more, was calculated with a 95% confidence interval (CI). *p*-values were calculated by Fisher’s exact test or the Wilcoxon rank sum test. A *p*-value < 0.05 was considered statistically significant.

## 3. Results

**Figure 1:**
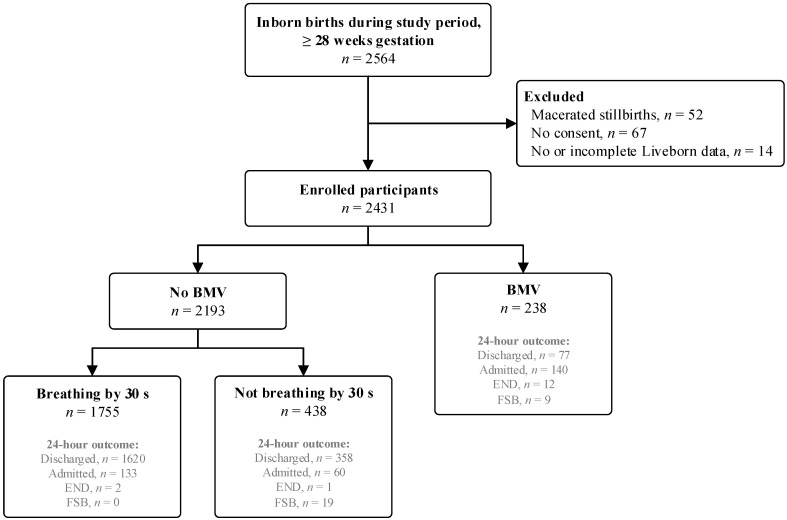
Flow diagram of enrolled participants. BMV = bag-mask ventilation, s = seconds, END = early newborn death, FSB = fresh stillbirth.

Over the study period, 2564 newborns of gestational age ≥28 weeks were born in the hospital. Of these, 2431 were enrolled in the study after the exclusion of 52 macerated stillbirths, 67 newborns who did not have parental consent to participate, and 14 cases with no or incomplete Liveborn app registrations. Of the enrolled newborns, 2193/2431 (90.2%) did not receive any BMV, whereas 238/2431 (9.8%) were ventilated. A flow diagram of study participants is shown in Figure 1. Among the newborns who started breathing without BMV, 193 newborns were admitted to the neonatal unit after birth, and 3 deaths were registered within 24 h. In 19 cases of fresh stillbirths, as classified by the midwives, the newborn never received any BMV attempt. Among those who received BMV, 12 (5.0%) newborns died within 24 h, and 9 (3.8%) were classified as fresh stillborn after attempted resuscitation.

Maternal and newborn characteristics for those who did not receive and did receive BMV are presented in Table 1. Newborns who received BMV were more often delivered by cesarean section.

### 3.1. Frequency, Sequence, Timing, and Duration of Interventions

Table 2 presents interventions after birth among newborns who did not receive BMV but started breathing before versus after 30 s and newborns who received BMV. Of the 2431 newborns observed, 1755 (72.2%) started breathing spontaneously by 30 s after birth, 438 (18.0%) started breathing after 30 s but did not receive BMV, and 238 (9.8%) were ventilated. Skin-to-skin contact was initiated for most newborns who initiated spontaneous breathing (77%), and 35% of those who received BMV experienced skin-to-skin contact before being moved to the resuscitation table. Almost all newborns were dried and stimulated immediately after birth. The number of stimulation sequences increased from a median of 1 (1, 1) among those breathing well by 30 s to 2 (2, 3) among those who received BMV, with a correspondingly longer total duration of stimulation, median 38 (24, 57) vs. 77 (49, 107) s. The median time to cord clamping was 120 (59, 166) for those breathing prior to 30 s, 124 (75, 182) s for those breathing after 30 s, and 31 (16, 79) s for those who received BMV. The use of suctioning increased from 9.5% to 32.9% to 66.8% across the three groups. The median time to start breathing after birth was 6 (3, 13) and 49 (38, 66) s in the before and after 30 s groups, respectively. For the BMV group, time to start BMV was 82 (54, 120) s, with 32% initiated within the first “golden” minute. The total duration of BMV was a median of 138 (85, 344) s, with a median of 2 (1, 3) number of ventilation sequences per newborn.

**Figure 2:**
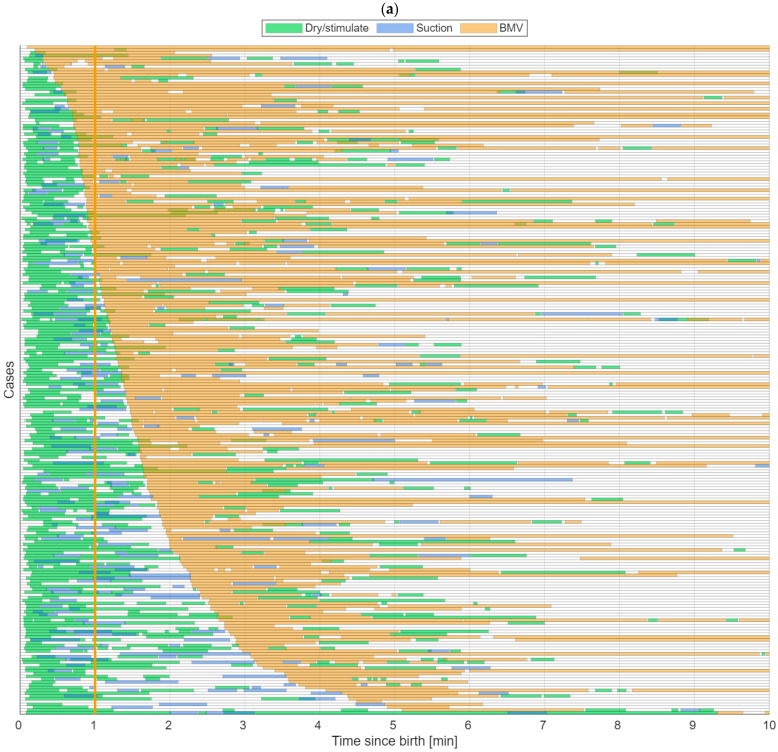

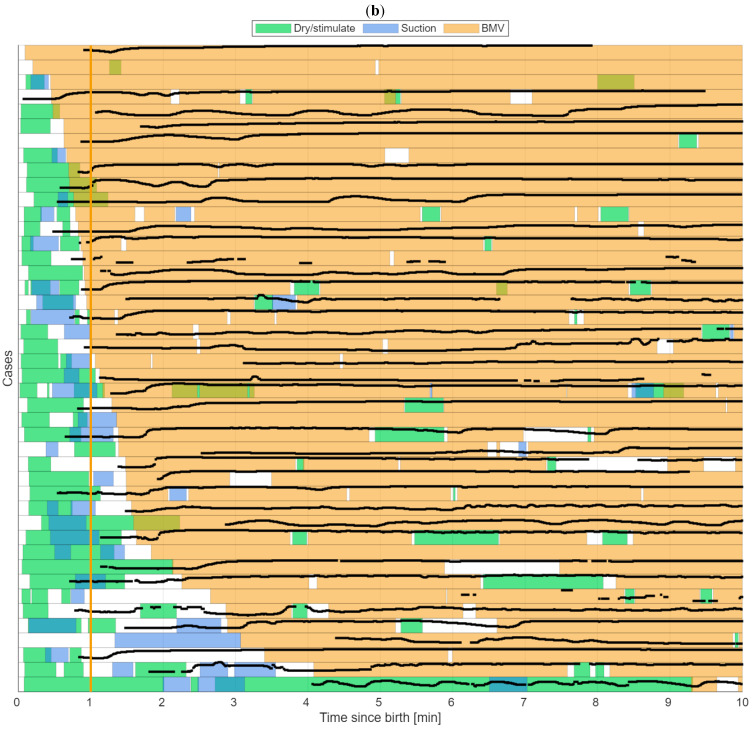
Frequency, sequence, timing, and duration of resuscitative interventions up to 10 min after birth among (**a**) the 238 newborns who received bag-mask ventilation (BMV) and (**b**) the 44 cases with prolonged BMV, the black lines represent heart rate.

Figure 2a illustrates the frequency, sequence, timing, and duration of resuscitative interventions up to 10 min from birth among the 238 newborns who received BMV. Figure 2b highlights the 44 cases with prolonged BMV (ongoing BMV at 10 min) and illustrates their heart rate changes. Nearly all cases were dried and stimulated extensively within the first minute. Suctioning was observed frequently throughout the early resuscitation period before starting BMV. Ventilations were continued without many interruptions (Figure 2a).

Each horizontal row represents one newborn. Zero is the time of birth. The orange vertical line indicates the “golden minute”. Drying/stimulation is shown in green, suctioning in blue, and ventilation in orange.

Heart rate is illustrated by the black line and is scaled from 0 to 200 beats/min with 100 beats/min in the middle of the box.

### 3.2. Newborn Outcomes in Relation to Duration of Ventilation

Table 3 presents the outcomes at 24 h and at 7 days after birth of the 238 newborns who received BMV in relation to the total duration of ventilation. Specifically, 36/238 (15.1%) newborns were ventilated for less than 60 s and no one died in this group. One fresh stillbirth was classified in this group after the short period of BMV. Additionally, 178/238 (74.8%) newborns were ventilated between 1 and 10 min, with 8 (4.5%) newborns dying within the first 24 h and 5 more within the next 6 days, leading to a total of 13 (7.3%) deaths within 7 days postpartum. Five fresh stillbirths were noted in this group. Among the 24 (10.1%) newborns who were ventilated for 10 min or more (≥600 s), 10 (41.7%) died within 7 days. The risk of dying within 24 h or 7 days increased significantly if BMV continued for more than 10 min with RR = 4.26, 95% CI; 1.3–10.0, *p* = 0.016 and RR = 8.14, 95% CI; 3.5–17.6, *p* < 0.001, respectively.

Three newborns were classified by the midwives as fresh stillbirths after more than 10 min of ongoing BMV.

### 3.3. Characteristics of Newborns Who Received Prolonged BMV

Prolonged BMV (ongoing at 10 min after birth) was registered in 44/238 (18.5%) newborns (Figure 2b). BMV was initiated within one minute after birth in 45.5% of these newborns, after short periods with stimulation and brief suctioning in some cases. The majority were ventilated continuously without interruptions throughout most of the resuscitation. Newborn heart rate recordings were visible in 38 cases, and in 3 of these cases the recordings were unstable (scattered line). Heart rate trajectories seem to vary throughout the 10 min of resuscitation, but an increase in heart rate was frequently observed after 30–60 s of ventilation. At 24 h after birth, 33/44 (75%) newborns were alive, whereas 11/44 (25%) had died. Maternal and newborn characteristics, labor and fetal heart rate information, resuscitation interventions, and newborn heart rate responses for these 44 cases, alive and dead, are presented in Table S1. Those who died within 24 h seemed to be more often admitted from another hospital with an abnormal fetal heart rate on admission. They were more often delivered by vacuum extraction or cesarean section with a final abnormal fetal heart rate recording before birth. Time to start BMV was longer among dead compared to alive newborns, 110 (56, 167) vs. 60 (42, 89) s, respectively (*p* = 0.09). Newborn heart rate at the start of BMV was similar among those who died and those still alive, 76 (70, 98) and 75 (61, 90) beats/min, respectively. Total duration of BMV was slightly longer among those who died, 645 (553, 836) versus 600 (549, 681) s among the alive, and at the end of BMV, heart rate had increased to 152 (110, 167) and 161 (153, 171) beats/min among those who died and the alive newborns, respectively. Approximately 2.5 min after discontinuation of BMV, the last recoded heart rate was significantly lower among those who died compared to those still alive at 24 h, 87 (75, 162) versus 158 (152, 170) beats/min, respectively (*p* = 0.04).

Video recordings were available for 21 of the 44 cases. Among these 21 newborns, 6 died within 24 h and 15 were alive. Measures to keep the baby warm were observed in 4/21 cases. Effective ventilation was observed in 11/21 cases, and 10 of these 11 newborns were alive at 24 h. In the other 10/21 newborns with ineffective ventilation, 7/10 seemed to have an overextended neck, and 5/10 (50%) newborns with ineffective ventilation died within 24 h. The evaluation of mask seal was difficult based on the video and was therefore not annotated.

Good team coordination was not observed in any of the resuscitations where the newborn died within 24 h.

## 4. Discussion

This study, from a referral hospital in a low-resource setting, provides a detailed description of initiation of newborn breathing and the frequency, timing, sequence, and duration of different interventions after birth. The majority of newborns (72%) initiated spontaneous breathing within 30 s, some (18%) started breathing beyond 30 s, while almost 10% received BMV. The initial steps of drying and stimulation were applied almost universally. Suctioning was practiced more frequently than expected according to recommendations, both among spontaneously breathing newborns and those who received BMV. Only one-third were ventilated within the “golden” first minute, and initiation of BMV was probably delayed in several cases due to excessive suctioning and stimulation. Ventilation was mostly conducted without many interruptions, but the risk of death within 24 h and 7 days increased more than four-fold and eight-fold, respectively, if ventilation was continued for more than 10 min.

### 4.1. Frequency, Sequence, Timing, and Duration of Interventions

Overall, we document a good adherence to essential newborn care guidelines like drying, keeping warm, stimulation, and early skin-to-skin contact among spontaneously breathing newborns. However, among newborns who were not breathing well by 30 s, the use of suctioning was likely unnecessarily high (33%). Suctioning is only recommended if the provider suspects an obstruction in the airway, hindering the flow of air, and excessive suctioning may be harmful [16,23,24]. Suctioning was also frequently used (67%) among the newborns who received BMV and was commonly applied before the start of ventilation. It is not recommended to suction before start of ventilation unless there are secretions obstructing the airway [23,24]. Among those receiving BMV, stimulation was frequently performed for a much longer time period than recommended. Excessive use of suction and stimulation likely delayed the initiation of BMV in many cases, as reported from other low-resource settings [16].

Only one-third of BMV was initiated within the so-called “golden minute” [6]. These findings point to persistent gaps in early recognition of and timely start of BMV among non-breathing newborns. Strengthening provider training and reinforcing adherence to neonatal resuscitation guidelines are critical steps to improve adherence to guidelines and improve newborn survival [18]. Graphical timelines, as presented in our study, may serve as a useful tool for reviewing complex newborn resuscitation situations and be used as a basis for continuous quality improvement efforts.

Almost 10% of the newborns in our population received BMV, and this proportion is higher than reported from similar settings in Tanzania [7,8] and in other settings [10,11,12,13,14,15,16]. However, 10% likely reflects the incidence of newborns in need of BMV in our study hospital. All newborns were closely observed by a trained observer, and no breathing efforts were registered before the start of BMV at a median of 82 s after birth. The first measured median heart rate of 96 beats/min was substantially lower in this group, receiving BMV, compared to those who did not receive BMV. Among those who did not receive BMV, 72% started breathing before 30 s, and as much as 18% started breathing after 30 s, but most of these were breathing within the first minute.

Based on our detailed and accurate data, we speculate that many newborns who would benefit from BMV do not receive this life-saving intervention in other similar low-resource settings.

### 4.2. Short Term Newborn Outcomes

Most newborns (75%) in our cohort were ventilated between one and 10 min, and 7.3% of these newborns died within 7 days postpartum. Among the 15% who were ventilated for less than one minute, no one died within 7 days. However, for those ventilated for more than 10 min (the remaining 10%) the risk of death increased significantly with more than a four-fold increased risk of death within 24 h and more than an eight-fold increased risk of death within 7 days as compared to those who were ventilated for less than 10 min. These findings are in line with a previous report from the same hospital, demonstrating a 6% increased risk of death and morbidity for every minute of applied BMV, adjusted for pregnancy and labor complications, birth weight, gestational age, and time to initiation of BMV [17].

On the other hand, among newborns who received an intermediate duration of BMV (i.e., 1–10 min) 78% were discharged at 7 days postnatal, indicating that adequate care was provided to this group.

A total of nine newborns were classified as fresh stillbirths by the midwives after attempting BMV. The HBB guidelines recommend starting BMV for all newborns who are not clearly identified as stillborn at birth, since it can be difficult to distinguish a true fresh stillborn from a severely asphyxiated newborn—the latter may survive adequate resuscitation [3,6,22,25,26].

In our study cohort, 44 newborns received prolonged BMV, i.e., were ventilated at 10 min after birth regardless of total duration of BMV. As many as 11 (25%) of these newborns died within 24 h, and several factors may have contributed to these deaths. They were often admitted from another hospital with an abnormal fetal heart rate on admission, and their first newborn heart rate was low, indicating that these newborns suffered from severe birth asphyxia. Importantly, the median time to start BMV among those who died was almost two minutes. This is substantially longer than recommended and almost twice as long as in the alive group, where time to start BMV was a median of one minute. A previous study, from the same hospital, has reported a 16% increase in death and morbidity for every 30 s delay in initiation of BMV, adjusted for pregnancy and labor complications, birth weight, and gestational age [17].

It is worth noting that BMV was delivered continuously without many interruptions, and for most newborns, the heart rate increased from the start of BMV to the end of BMV to a median of 152 beats/minute among those who died and 161 beats/minute among alive newborns. However, after discontinuation of BMV, heart rate decreased rapidly among those who died. We suspect that little attention to keeping the newborn warm, ineffective ventilation, and poor team coordination, as observed by video, partly contributed to several of these deaths. Furthermore, post-resuscitation care may have been suboptimal.

### 4.3. Strengths and Limitations

The strength of this study is the unique comprehensive research infrastructure with trained data collectors observing all deliveries 24 h a day, 7 days a week, using the Liveborn Observation app for real-time registration of events. Video cameras were installed in all five labor rooms and in the operating theater. Thus, the study offers objective insight into real-world resuscitation practices and areas where targeted improvement efforts may yield significant clinical benefit.

The study has some important limitations. First, the study period is limited to eight and a half months, and potential seasonal variation is not captured. Second, some Liveborn registrations may suffer from incorrect annotation and timing. Third, we only had adequate video recordings of half of the cases with prolonged BMV, and the video quality and camera positioning restricted visibility of some clinical actions. Fourth, the findings reflect practices in a single center and may not be generalizable to other facilities with different resources, staffing, or training backgrounds. Fifth, the behavior of healthcare providers may have been influenced by the presence of video cameras and research observers. Sixth, this study did not employ multivariable modeling to control for potential confounding variables, and the observed associations between duration of BMV and deaths should be interpreted with caution. Seventh, heart rate data were missing for a proportion of cases. Finally, the conclusion is limited by the lack of assessment of the quality of ventilations provided.

Despite these limitations, the consistent patterns observed align with prior studies and highlight critical points for intervention. While the single-site design may limit generalizability, the site’s characteristics and the study’s rigorous methodology support the transferability of findings to comparable settings.

## 5. Conclusions

In summary, approximately one in ten newborns required BMV at birth, but only one-third received ventilation within the first minute as recommended by HBB guidelines. While most newborns responded promptly to ventilation, prolonged BMV beyond 10 min was associated with higher mortality. Suboptimal adherence to resuscitation protocols, particularly excessive use of stimulation and suction, delays in initiating ventilation, and inconsistent ventilation quality, highlight areas for continued quality improvement in neonatal resuscitation practices.

## Figures and Tables

**Table 1:** Demographic characteristics of 2431 enrolled participants who received bag-mask ventilation (BMV) and not.

Characteristics	No BMV *n* = 2193	BMV *n* = 238
*Maternal age*, *n* (%)		
˂20	309 (14.1)	42 (17.6)
20–34	1544 (70.4)	156 (65.5)
>34	340 (15.5)	40 (16.8)
Missing	0	0
*Parity*, *n* (%)		
0	687 (31.3)	86 (36.1)
1–2	781 (35.6)	79 (33.2)
>2	725 (33.1)	73 (30.7)
Missing	0	0
*Birth weight* (g)		
<1500	17 (0.8)	8 (3.4)
1500–2499	136 (6.2)	22 (9.2)
2500–3999	1954 (89.1)	195 (81.9)
≥4000	85 (3.9)	12 (5)
Missing	1 (0)	1 (0.4)
*Gestational age* (weeks)		
28–33	40 (1.8)	13 (5.5)
34–36	151 (6.9)	9 (3.8)
37–41	1806 (82.4)	194 (81.5)
≥42	73 (3.3)	8 (3.4)
Missing	123 (5.6)	14 (5.9)
*Multiplicity*, *n* (%)		
Singleton	2130 (97.1)	229 (96.2)
Twins	62 (2.8)	8 (3.4)
Triplets	1 (0)	1 (0.4)
Missing	0	0
*Newborn sex*, *n* (%)		
Male	1135 (51.8)	137 (57.6)
Female	1058 (48.2)	101 (42.4)
Ambiguous	0	0
Missing	0	0
*Mode of Delivery*, *n* (%)		
Vaginal delivery	1682 (76.7)	101 (42.4)
Vacuum extraction (vacuum)	48 (2.2)	21 (8.8)
Vaginal breech delivery	11 (0.5)	13 (5.5)
Cesarean section	452 (20.6)	103 (43.3)
Missing	0	0

**Table 2:** Interventions after birth among newborns not receiving bag-mask ventilation (BMV) who were breathing well or not breathing well by 30 s and newborns receiving BMV.

	No BMV		BMV
Interventions After Birth	Breathing Well by 30 s *n* = 1755	Not Breathing Well by 30 s *n* = 438	*n* = 238
*Skin-to-skin contact*, *n (%)*	1344 (76.6)	339 (77.4)	84 (35.3)
Initiation time from birth, sec	38 (27, 53)	42 (29, 61)	35 (28, 51)
Missing annotations, *n* (%)	411 (23.4)	99 (22.6)	154 (64.7)
*Drying/stimulation*, *n* (%)	1742 (99.3)	427 (97.5)	235 (98.7)
Initiation time from birth, sec	5 (4, 9)	5 (3, 9)	5 (3, 8)
Number of sequences per newborn	1 (1, 1)	1 (1, 2)	2 (2, 3)
Total duration, sec	38 (24, 57)	56 (36, 81)	77 (49, 107)
Missing annotations, *n* (%)	13 (0.7)	11 (2.5)	3 (1.3)
*Cord clamp*, *n* (%)	1742 (99.3)	431 (98.4)	231 (97.1)
Initiation time from birth, sec	120 (59, 166)	124 (75, 182)	31 (16, 79)
Missing annotations, *n* (%)	13 (0.7)	7 (1.6)	7 (2.9)
*Suction*, *n* (%)	167 (9.5)	144 (32.9)	159 (66.8)
Initiation time from birth, sec	44 (22, 79)	32 (20, 58)	44 (24, 78)
Number of sequences per newborn	1 (1, 1)	1 (1, 1)	1 (1, 2)
Total duration, sec	27 (16, 44)	29 (17, 53)	26 (17, 40)
*Time to start breathing*, s	6 (3, 13)	49 (38, 66)	162 (46, 288)
*First newborn heart rate*, bpm	170 (153, 186)	154 (131, 173)	96 (71, 137)
Missing, *n* (%)	445 (25.5)	115 (26.3)	41 (17.2)
*Bag-mask ventilation*, *n* (%)	0	0	238 (100)
Initiation time from birth, sec	0	0	82 (54, 120)
BMV initiated prior to 60 s, *n* (%)	0	0	76 (31.9)
Number of sequences per newborn	0	0	2 (1, 3)
Total duration, s	0	0	138 (85, 344)

Data is shown as *n* (%) and median (quartiles 1, 3). bpm = beats per minute.

**Table 3:** Outcome at 24 h and 7 days after birth in relation to total duration of bag-mask ventilation (BMV).

Characteristics	Outcome at 24 h *n* = 238	Outcome at 7 Days *n* = 238
*Duration of BMV 1–60 s*	36 (15.1%)	36 (15.1%)
Discharged	22 (61.1%)	34 (94.4%)
Admitted to neonatal unit	13 (36.1%)	1 (2.8%)
Dead	0 (0.0%)	0 (0.0%)
Fresh stillbirth	1 (2.8%)	1 (2.8%)
*Duration of BMV 61–599 s*	178 (74.8%)	178 (74.8%)
Discharged	55 (30.9%)	138 (77.5%)
Admitted to neonatal unit	110 (61.8%)	22 (12.4%)
Dead	8 (4.5%)	13 (7.3%)
Fresh stillbirth	5 (2.8%)	5 (2.8%)
*Duration of BMV ≥ 600 s*	24 (10.1%)	24 (10.1%)
Discharged	0 (0.0%)	6 (25.0%)
Admitted to neonatal unit	17 (70.8%)	5 (20.8%)
Dead	4 * (16.7%)	10 (41.7%)
Fresh stillbirth	3 (12.5%)	3 (12.5%)

* 3 deaths were registered within 30 min of birth.

## Data Availability

The data supporting the findings of this study are available from the corresponding author upon reasonable request. Due to regulations established by the National Institute for Medical Research (NIMR) in Tanzania, these data cannot be made publicly accessible.

## Supplementary Materials

The following supporting information can be downloaded at https://www.mdpi.com/article/10.3390/children13050614/s1. Figure S1: Overview of the resuscitation station (called infant warmer) equipped with an overhead warmer, suction device, NeoBeat newborn heart rate monitor, Upright Resuscitator, and a tablet with video camera (covering the newborn and hands of healthcare providers) and display. The tablet connects with the Liveborn Observation app. Figure S2: Illustration of the newborn resuscitation station with a midwife simulating a newborn resuscitation, observed by a trained data collector who is recording interventions in real-time using the Liveborn Observation app. Table S1: Maternal and newborn characteristics, labor and fetal heart rate information, resuscitation practices, and newborn heart rate responses among newborns who received ventilation at 10 min after birth.

## Abbreviations

BMV	bag-mask ventilation
HBB	Helping Babies Breathe
WHO	World Health Organization
