# Supplementary material for: Newborn Resuscitation Practices and Outcomes in Rural Tanzania—A Real-Time Observational and Video Study
Source: Children (Basel). 2026 Apr 28;13(5):614. doi: 10.3390/children13050614 (PMC13204974; doi:10.3390/children13050614)
Supplement: Supplementary file 1 [file children-13-00614-s001.zip › children-4203931-supplementary.pdf]

Figure S1. Overview of the resuscitation station (called infant warmer) equipped with overhead warmer, suction device, NeoBeat newborn heart rate monitor, Upright Resuscitator, and a tablet with video camera (covering the newborn and hands of healthcare providers) and display. The tablet connects with the Liveborn Observation app

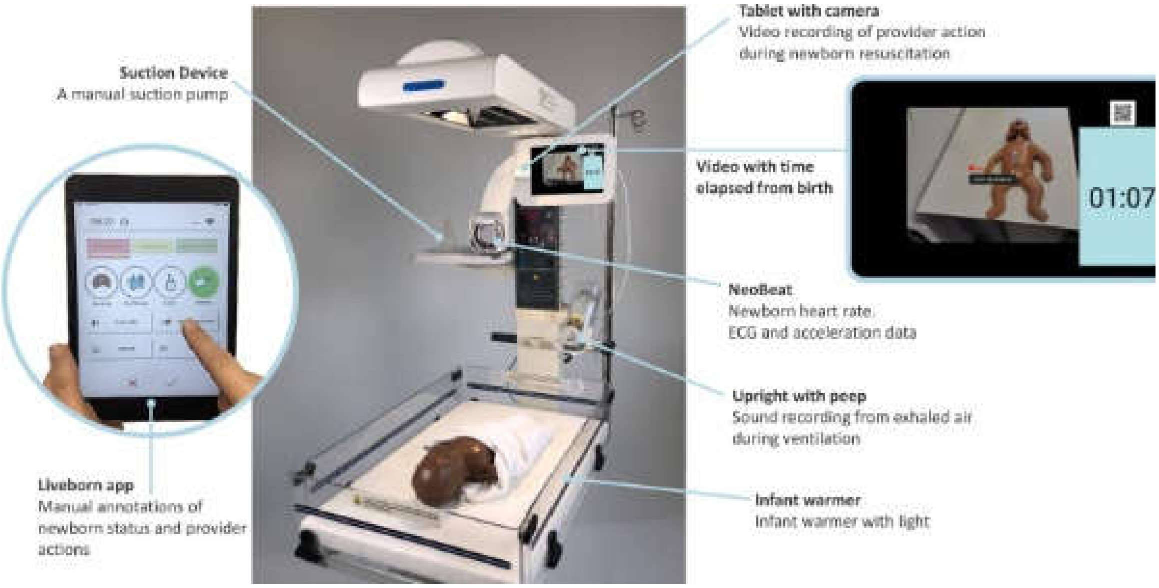

Figure S2: Illustration of the newborn resuscitation station with a midwife simulating a newborn resuscitation, observed by a trained data collector who is recording interventions in real-time using the Liveborn Observation app.

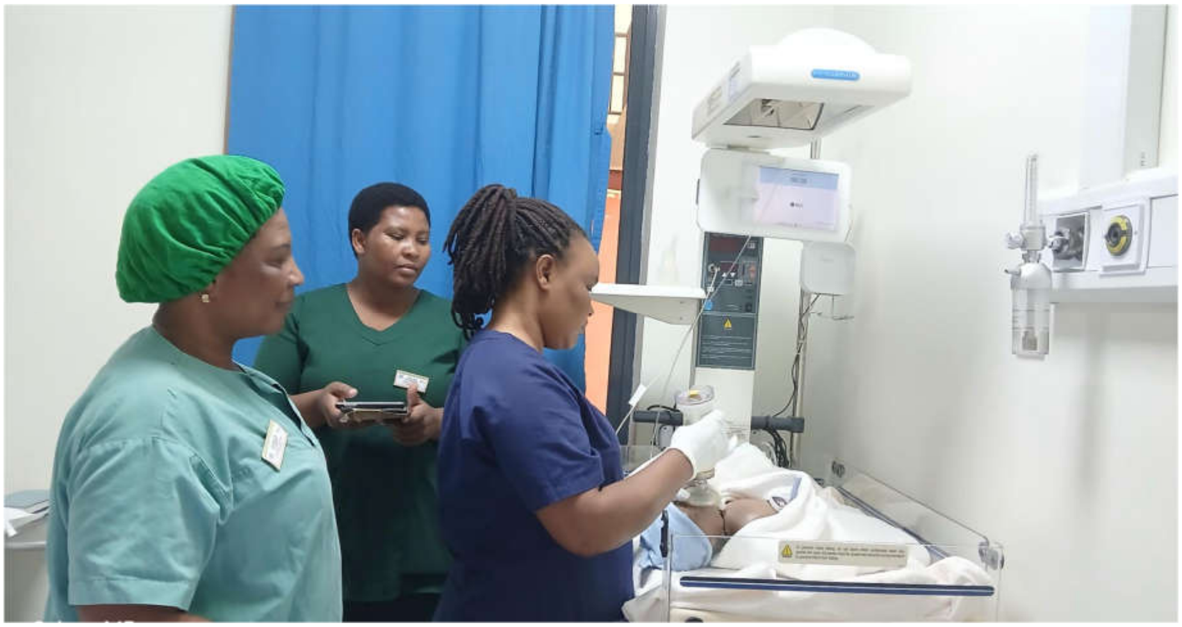

Følsomhet Intern (gul)

Table S1. Maternal and newborn characteristics, labour and fetal heart rate information, resuscitation practices, and newborn heart rate responses among newborns who received ventilation at 10 min after birth.

	**All**<br>**N = 44**	**Alive (24-hour)**<br>**N = 33**	**Dead* (24-hour)**<br>**N = 11**
**Baseline characteristics**			
**Source of admission**			
Other health facility	7 (15.9)	4 (12.1)	3 (27.3)
Home	30 (68.2)	24 (72.7)	6 (54.6)
Antenatal room	7 (15.9)	5 (15.2)	2 (18.2)
**Maternal age**			
<20	11 (25)	9 (27.3)	2 (18.2)
20-34	26 (59.1)	19 (57.3)	7 (63.6)
>34	7 (15.9)	5 (15.2)	2 (18.2)
**Parity**			
0-2	25 (56.8)	20 (60.6)	5 (45.5)
>2	19 (43.2)	13 (39.4)	6 (54.5)
**Birth weight** (grams)			
<1500	1 (2.3)	0 (0.0)	1 (9.1)
1500-2499	6 (13.6)	5 (15.2)	1 (9.1)
2500-3999	35 (79.5)	28 (84.8)	7 (63.6)
≥4000	2 (4.5)	0 (0.0)	2 (18.2)
**Gestational Age** (weeks)			
28-33	2 (4.5)	0 (0.0)	2 (18.2)
34-36	2 (4.5)	2 (6.1)	0 (0.0)
37-41	32 (72.7)	25 (75.8)	7 (63.6)
≥42	4 (9.1)	3 (9.1)	1 (9.1)
Missing	4 (9.1)	3 (9.1)	1 (9.1)
**Multiplicity**			
Singleton	42 (95.5)	32 (97.0)	10 (90.9)
Twins	2 (4.5)	1 (3.0)	1 (9.1)
Triplets	0 (0.0)	0 (0.0)	0 (0.0)
**Newborn Sex**			
Male	24 (54.5)	18 (54.5)	6 (54.5)
Female	20 (45.5)	15 (55.5)	5 (45.5)
Ambiguous	0 (0.0)	0 (0.0)	0 (0.0)
**FHR on admission**			
Normal	40 (90.9)	31 (94.0)	9 (81.8)
Abnormal	3 (6.8)	1 (3.0)	2 (18.2)
Not measured	1 (2.3)	1 (3.0)	0 (0.0)
**Devices used to monitor FHR**			
Pinard	14 (31.8)	11 (33.3)	3 (27.3)
Moyo	27 (61.4)	21 (63.6)	6 (54.5)
Doppler	1 (2.3)	0 (0.0)	1 (9.1)

Følsomhet Intern (gul)

None	1 (2.3)	1 (3.0)	0 (0.0)
Missing	1 (2.3)	0 (0.0)	1 (9.1)
**Final FHR before birth**			
Normal	37 (84.1)	29 (87.9)	8 (72.7)
Abnormal	6 (13.6)	3 (9.1)	3 (27.3)
Not detected	0 (0.0)	0 (0.0)	0 (0.0)
Not measured	1 (2.3)	1 (3.0)	0 (0.0)
**Time of final FHR measurement before birth**			
1-5 minutes	17 (31.8)	14 (42.4)	3 (27.3)
6-15 minutes	8 (18.2)	5 (15.2)	3 (27.3)
16-124 minutes	18 (40.9)	13 (39.4)	5 (45.5)
Not measured	1 (2.3)	1 (3.0)	0 (0.0)
**Mode of Delivery**			
Vaginal delivery	18 (40.9)	16 (48.5)	2 (18.2)
Vacuum extraction	5 (11.4)	3 (9.1)	2 (18.2)
Vaginal breech delivery	5 (11.4)	5 (15.2)	0 (0.0)
Cesarean section	16 (36.4)	9 (27.3)	7 (63.6)
**Resuscitative interventions**			
Time to first stimulation, seconds	5 (3, 9)	7 (5, 9)	4 (3, 7)
Total stimulation time, seconds	61 (34, 85)	62 (38, 101)	61 (29, 74)
Time to first suction, seconds	30 (21, 48)	28 (17, 56)	33 (27, 41)
Total suction time, seconds	29 (19, 42)	30 (23, 38)	21 (17, 54)
Time to start BMV, seconds	66 (47, 97)	60 (42, 89)	110 (56, 170)
Total BMV time, seconds	610 (551, 732)	600 (549, 681)	654 (553, 836)
**Newborn heart rate**			
Time to first HR, seconds	67 (47, 91)	55 (46, 89)	72 (49, 206)
First HR, bpm	82 (69, 101)	85 (64, 102)	76 (69, 101)
HR at start BMV, bpm	76 (64, 90)	75 (61, 90)	76 (70, 98)
Missing	21	16	5
HR at end BMV, bpm	161 (149, 169)	161 (153, 171)	152 (110, 167)
Missing	16	10	6
Last HR recorded, bpm	156 (140, 166)	158 (152, 170)	87 (75, 162)
Time of last HR, seconds	766 (685, 858)	759 (691, 902)	808 (650, 830)

*Fresh stillbirths as classified by the midwives are included. Data is shown as n (%) and median (quartiles 1, 3).

FHR = fetal heart rate, BMV = bag mask ventilation, HR = heart rate, bpm = beats per minute,
